# Ontario’s Bad Example Continues

**Published:** 1898-04

**Authors:** 


### ONTARIO'S BAD EXAMPLE CONTINUES.

WE wish to extend to the veterinarians of Ontario our most sincere and heartfelt sympathy. It is the function of veterinarians not only to cure diseases, but to prevent them, to the greatest possible extent; and it is for the purpose of avoiding loss from disease and protecting the livestock industry that veterinary schools have been established and are supported by so many enlightened governments. It is recognized almost universally—the exceptions being Ontario and a few other places—that matters relating to the care and preservation of the health of animals, and especially when it is desired to avoid the effects of any infectious disease, should be planned and executed by veterinarians. Measures for the extermination of a pestiferous insect must be directed by an entomologist; the matter of improving the orchards of a State must necessarily be under the care of horticulturists. If the Government desires an investigation of the mines of a region it employs skilful and experienced mining-engineers. If material for an engine, gun, or vessel is to be examined and tested, it is sent to a suitable laboratory where the work can be carried out by mechanical engineers, metallurgists, chemists, or other experts in the case in point. But when it comes to tuberculosis of cattle, Ontario disregards all of these well-established precedents and principles, and appoints a certain lieutenant-colonel to instruct the farmers how to make tests of their own cattle, and apparently does not realize the inevitably disastrous result of this procedure.

We have the kindest feelings for our Canadian neighbors. We realize that many of the best-bred and most perfect specimens of nearly all breeds of domestic animals to be found in North America are in Canada. The annual fair at Toronto brings together the best collection of pure-bred livestock that can be found on this side of the Atlantic Ocean. We appreciate the benefits that the livestock interests of the United States have derived from the infusion of the fresh and high-class blood of Canadian animals. The Canadian breeders possess in a very high degree the inborn love for animals, and the natural and acquired aptitude for maintaining breeds that are so rare even among handlers and breeders of livestock. So it is probable that the United States will need to continue for many years to draw upon the livestock of Canada for improved, high-class animals. For this reason the action of the Canadian authorities in dealing with tuberculosis of cattle is of the highest importance to everybody interested in the improvement and health of the cattle of the United States. Many of our Eastern States now have laws providing for the inspection of cattle with tuberculin before they can be admitted into those commonwealths. A tuberculin-test of cattle coming from Canada is also required by regulation of the Bureau of Animal Industry. A large and constantly increasing number of our most progressive breeders require a test with tuberculin before breeding animals can be added to their herds. There must be a reason for these regulations. It is that tuberculosis has caused enormous losses in some of our best herds. Breeders have learned not only to fear it, but they have learned how to guard against its ravages, namely, by starting with healthy cattle and admitting to their herds only such cattle as have been proven to be free from tuberculosis. Can purchasers of Canadian cattle have this guarantee? Evidently not. It is well known that cattle that have been tested with tuberculin frequently fail to react upon a second test, although they may have presented a high and characteristic reaction at the first test. Now, if the Government of Ontario is to encourage, as it is unquestionably doing, the general and indiscriminate use of tuberculin by men who have absolutely no knowledge of this substance or of diseases, it must be clear that tuberculin-tests applied to Ontario cattle must have but little value. Again, what is done with the cattle that are condemned in these unofficial tests? Echo answers—What? Sold for export to the United States?

In the last number of the Ontario *Agricultural Gazette*, which is described as the official bulletin of the Farmers' Institute system of the Province of Ontario, there appears a little article on tuberculin by the bacteriologist of the Ontario Agricultural College at Guelph. It appears from this article that tuberculin is made at the Agricultural College and sent out to applicants in Ontario. It is supplied to farmers desirous of testing their own cattle, free of charge, while veterinarians are discriminated against, and are required to pay ten cents per dose. It is also stated that although reports are desired, no name or address is asked for, simply the record of the test. It is indeed strange that the Ontario authorities cannot see what must be perfectly clear to all who have had experience with this disease, namely, that the method adopted by them is sure to bring the tuberculin-test into unjust disrepute in Ontario, will injure confidence in and the reputation of Ontario cattle abroad, and must in the end work material injury to the livestock interests of that country. What, then, is the purpose of this exceedingly remarkable procedure? No good can come from it, much injury must result from it, and the only possible, and perhaps the most charitable, explanation that appears upon the surface, is that a certain lieutenant-colonel wanted a position, and had influence enough to get one at any cost.

